# Association between Handgrip Strength, Skinfold Thickness, and Trunk Strength among University Students

**DOI:** 10.3390/diagnostics13050904

**Published:** 2023-02-27

**Authors:** Snehil Dixit, Kumar Gular, Ajay Prashad Gautam, Ravi Shankar Reddy, Irshad Ahmad, Jaya Shanker Tedla, Vani Taneja

**Affiliations:** 1 Department of Medical Rehabilitation Sciences, College of Applied Medical Sciences, King Khalid University, Abha 61421, Saudi Arabia; 2 Department of Paediatric Dentistry, Batterjee Medical College, Jeddah 21442, Saudi Arabia

**Keywords:** young adults, hand grip strength, T score, skinfold thickness, trunk strength

## Abstract

Objectives: The main goal was to explore the relationship between hand grip strength (HGS), the thickness of the skinfold at multiple sites, and the trunk flexor (TF) and extensor (TE) muscle strength among healthy participants. Methods: We employed a cross-sectional design and randomly recruited 40 participants. Ultimately, only 39 participants were included. First, measurements for demographic and anthropometric variables were carried out. After that, the evaluation of hand grip strength and skinfold was performed. Data Analysis: Descriptive statistics were used to explore the amount of interaction between the smoking and nonsmoking groups, and a repeated measures analysis of variance was employed. Furthermore, associations between dependent and independent variables were discovered through a multiple linear regression model. Results: The participants had a mean age of 21.59 ± 1.19 years. The results of the repeated measures analysis of variance validated an acceptable interaction between the trunk and hand grip strength at a significance level of *p* < 0.01, further emphasized by their moderate association (*p* < 0.05). Multiple regressions between TE, TF, the independent variables T score, height, and age were also significant (*p* < 0.05). Conclusions: The trunk muscle strength can be used as a health indicator for comprehensive evaluation. The present study also found a moderate relationship between hand grip strength, trunk strength, and T score.

## 1. Introduction

In recent times, handgrip strength (HGS) has proven to be an ideal indicator for all-cause mortality and morbidity [1]. Currently, HGS is considered one of the most widely used comprehensive measures globally by clinicians and social agencies, as the evidence supports the measure as one of the best health indicators [2]. Muscle strength is imperative for daily functions and health—any decrease in strength may adversely affect health as well. In addition, muscle strength has been associated with health status irrespective of age and clinical condition [3]. 

HGS has been labeled as a biosignature for health [4]. There is already ample evidence that supports the current proposition. The evidence includes reliable data showing that HGS can be an explanator for health that coincides with a variety of conditions, such as bone mineral density, diabetes, multimorbidity, fractures, the outcome of surgical interventions, falls, depression, cognitive impairment, and quality of life [4,5]. In addition, a predictive association between HGS and mortality, morbidity, and problems related to frequent hospitalization can also be found [4].

So far, a wide range of research has documented that a decrease in grip strength may be linked with a hike in the disease and death rate, longer durations of hospital stays, frequent falls, malnourishment, and poor quality of life [2,6]. In addition, similar relationships have been detected in the general and specific populations with the disease. 

In a study aimed at investigating the relationship between HGS and the hazard of significant causes of mortality, a low level of HGS proved to be one of the factors related to a high disease incidence and mortality rate due to cardiovascular disease (CVD) in the hypertensive population [7]. Additionally, the data released by the UK biobank, with a sample size of 502,628 adult individuals and a follow-up of 7.1 years, revealed that for every decrease of 5 kg force in HGS, there was a considerable upswing in mortality and death from CVD [8].

Sarcopenia may be associated with weak HGS, low muscle mass, or a slow walking speed [9]. Typically, Irisin (IR), formed by adipose tissue and muscle, is found at lower concentrations among people suffering from sarcopenia [9]. The HGS may be further influenced by forearm circumference, palm circumference, wrist joint circumference, and hand length [10].

Trunk endurance and strength are essential parameters for day-to-day functioning. They are also a measure of physical fitness [11]. The trunk muscles are active during sitting, standing, lifting weights, and balancing oneself in dynamic situations. Day-to-day activities and optimum health demand the ideal muscle endurance and strength of the trunk [6,11]. Both muscular strength and endurance are necessary for recreational activities and daily task performance [11]. Physiologically, the trunk muscles are more suitable for low levels of training for more extended periods. In addition, static and dynamic activities can be well-coordinated when the trunk muscles are in a healthy state [11]. Studies have also indicated that weak trunk muscle strength is associated with greater chances of falls and related complications [12]. Although it has been observed that trunk muscle strength is associated with musculoskeletal problems, the confirmatory role of trunk muscle strength in other health issues still needs to be explored. 

On the contrary, skinfold thickness measurements are conventionally used to classify an individual in terms of relative fat or to evaluate specific subcutaneous tissue fat. Usually, the measures are swift and uninvasive, allowing one to obtain raw data from the population in a wide range of age groups. Moreover, the readings are generally reliable and valid, with low intra-observer and inter-observer errors [13]. Waist circumference (WC) also provides a simple yet precise method to measure central fat. Usually, it is observed that WC may be more associated with adverse outcomes such as an abnormal lipid profile or insulin resistance; hence, it is vital to include it in clinical studies. Research examining the relationship between WC and abdominal fat with the help of magnetic resonance imaging (MRI) has also revealed consistent correlations in the range of 0.5 to 0.8, showing it to be an effective anthropometric measure [13]. 

Previous literature has indicated a strong association between hand grip strength and the upper body, neck musculature, and neck circumference [11,14]. On the other hand, research exploring the association between HGS, skinfold thickness (multiple sites), and trunk strength is limited. However, no data are available among the young population to explore the relationship between hand grip strength, skinfold measurement (multiple sites), and trunk muscle strength (flexor and extensors). This may help in establishing how skinfold thickness influences the muscle functions, i.e., trunk muscle and hand muscle strength. This knowledge could help clinicians to plan interventions targeting not only an improvement in muscle strength but also taking into consideration the skinfold thickness. Hence, the main objective of the present study was to discover the association between hand grip strength, trunk muscle strength, and skinfold thickness at multiple sites in healthy participants.

## 2. Methods

Before the study’s commencement, ethical clearance was obtained under number ECM#2021-3603, and we registered with clinicalTrials.gov using the identifier NCT05231291. Consent forms were obtained from the participants before the test enrollment. The study sample was determined using the sample included in another study [15] in the same population. The sample totaled 74 for the age group 18–29. However, due to constraints imposed by COVID-19, we achieved a final sample of 39, as one of the participants and the evaluator both tested positive for COVID-19. In this cross-sectional study, 39 male students between 18 and 24 years, with a mean age (years) of 21.59 ± 1.19, were randomly selected using probability sampling from the college student registry of the university (male section). Only healthy participants with no known history of previous diseases were included (*n* = 39). Students who found it difficult to follow the tests’ instructions after one familiarity session were excluded (*n* = 0). Students reporting acute infections and a previous history of critical respiratory illness were also excluded (*n* = 1). 

### 2.1. Measurements

COVID-19-related guidelines were followed before the commencement of measurements [16]. Baseline measurements for age in years, smoking history, and standing height (cm) were also calculated. Waist circumference measurements strictly adhered to the recommendations set by the World Health Organization (WHO) [17].

Three evaluators assessed the trunk muscle strength, HGS, and skinfold thickness (SFT), respectively. The muscles chosen for measuring SFT were the biceps, triceps, and subscapular and suprailiac muscles on the side that was non-dominant [18,19]; the measurements were carried out by a well-trained investigator at various sites marked using a Harpenden caliper (Baty, Burgess Hill, UK). The instruments were well-calibrated before testing. Skinfold thickness was measured for each muscle from at least two different sites after marking. A third measurement was compulsory if the smallest distance for the estimated sites exceeded 3 mm. The body fat percentage was obtained by inserting the mean of the two closest measurements into the formula provided by Durnin and Womersley [20,21]. Siri’s equation (([4.95/BD] − 4.50) × 100) was used to attain the percentage of fat mass (%) from the percentage of body fat [21]. The fat mass (FM, kg) and fat-free mass (FFM, kg) were computed as (%F × weight)/100 and weight − FM, respectively. 

### 2.2. Hand Grip Strength Measurement 

Jamar Hydraulic Dynamometers were used to measure HGS (Hydraulic Hand Dynamometer Fabrication Enterprises Inc., New York, NY, USA). The investigators ensured that the Jamar handgrip dynamometer was functional and attuned before the measurements. Furthermore, the procedures and instructions for measuring the participants’ handgrip strength obeyed the National Institute of Health Research (NIHR) recommendations [22].

Before the actual trial, the contributors to the research were shown how to use the dynamometer. The standard operating procedures ensured that participants sat comfortably with a good posture of the spine, the forearm in a mid-prone position, ample hand support, the wrist free from the chair’s armrest, and the thumb pointing up. Additionally, before each measurement, we made sure that the needle in the dial of the dynamometer was at the zero position. 

The HGS measurement was first carried out on the right side, followed by the left side. Standard phrases were used to encourage the participants to squeeze for longer to obtain the best possible results until the needle stopped rising. The test was disregarded and repeated if the participant’s arm was elevated above the arm of the chair or if the feet were lifted off the ground during the test. Three alternate measurements were recorded for each hand. 

The formula used to attain the T score for hand grip strength is as follows [23]:

T score = (Grip strength_Participant_ − Grip strength_Young adult mean_)/Standard deviation (SD)_Young adult mean_

Grip strength was designated as low if the acquired T score was either equivalent or two points less than the established age- or gender-matched lower levels for grip strength [23,24,25]. Moreover, the mean predicted HGS for a given height was calculated. 

The formula used to predict the hand grip strength according to the participant’s height was as follows:

[Measured height − Mean young height] × ꞵ + mean young HGS—the regression slope between HGS and height considered as “ꞵ” beta.

### 2.3. Trunk Muscle Strength Testing Procedure

An appropriate washout period was provided before the performance of the test. An analogue hydraulic push–pull dynamometer (model: FEI-12-0394) was used for evaluation. First, the trunk flexor and extensor muscle vigor was assessed by means of a dynamometer and documented in pounds (lbs). The reproducibility and appropriateness of the dynamometer have already been established in earlier studies, with intra-class correlation coefficient values greater than 0.940 [4]. Trunk flexor strength was evaluated by half lying with the trunk supported, 30° of hip flexion, and knees in full extension. Meanwhile, the hands were crossed and placed above the acromion process.

In comparison, extensor muscle endurance was evaluated by prone lying with the hip at 30° flexion, stabilizing the posterior superior iliac spine, and placing the dorsal aspect of the hand over the forehead. Finally, the dynamometer was applied perpendicularly below the suprasternal notch and at the T4 spinous process to measure trunk flexor and extensor strength. An initial trial test was followed by three measurements exerting force against the dynamometer for 3–5 s, and the mean of the three trials was calculated and recorded.

## 3. Data Analysis

The descriptive statistics used to exhibit quantitative and qualitative variables were average and standard deviations and frequencies. In addition, fat status categorization and categorical frequency data calculations were also executed. 

Next, the variations that occurred while performing multiple measurements were investigated using a one-way repeated analysis of variance (RANOVA). To explore the amount of interaction between the smoking and nonsmoking groups, RANOVA was used. If the assumption for Mauchly’s test was violated, then Greenhouse–Geisser values were used for interpretation, and the *p*-value for significance was fixed at *p* < 0.05 (95% confidence interval). RANOVA was performed for the primary outcomes using statistical package for the social sciences (SPSS). Further, the results of the RANOVA were calculated as degrees of freedom (Df1, Df2) and F and *p* values.

The amount of interaction between anthropometric characteristics and HGS was analyzed using the Pearson correlation coefficient. Further, associations between dependent and independent variables were discovered through a multiple linear regression model. Using the stepwise method, multiple regression analysis was used to evaluate likely influencers of HGS. The degrees of freedom (Df), *p*-value, F value, and R^2^ values were calculated for the regression analysis. In the present study, a *p*-value less than 0.05 was considered significant. All parameters are reported per the international system of units (SI). 

## 4. Results

**Figure 1:**
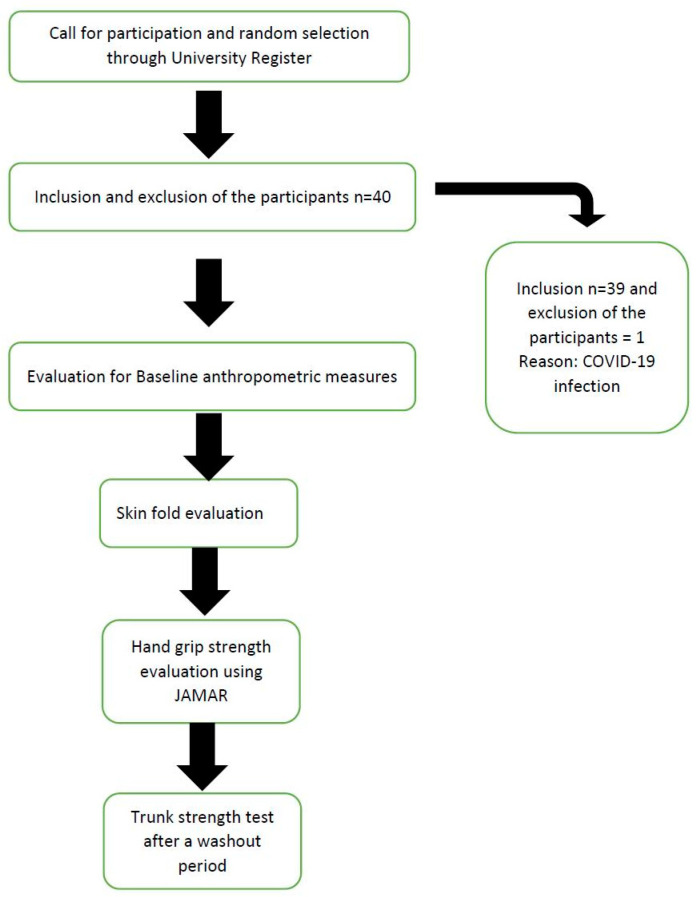
Recruitment flow of the participants in the study.

**Figure 2:**
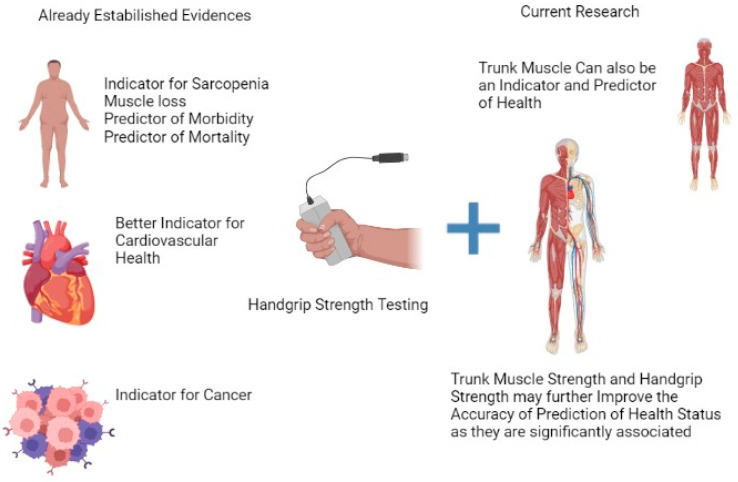
Summary of the clinical utility of the present evidence.

Ultimately, *n* = 39 university students with a mean age of 21.59 ± 1.19 met the selection criteria. The demographic and anthropometric details of the participants are presented in Table 1. The distribution of participants according to body fat percentage is described in Table 2, and the T scores of HGS are shown in Table 3. Figure 1 displays the flow of participants, and Figure 2 summarizes the clinical utility of the measures. 

The RANOVA statistical tool was used to assess the interaction between smokers and nonsmokers. The assumption for Mauchly’s test was violated; therefore, Greenhouse–Geisser values were used for interpretation, and the *p*-value for significance was set at *p* < 0.05. The two factors that were considered to create the RANOVA model were HGS (left and right) and fat percentage. The age, waist circumference, and other factors did not show any traceable interaction between the smokers and the nonsmokers. The other two factors considered for the model were trunk strength for extensors and flexors (TE and TF) and HGS (right and left). The interaction effect for HGS (left and right) and the trunk strength test (TE and TF) were analyzed using RANOVA considering within-subject factors such as waist circumference and smoking habits among the participants. As the assumption for Mauchly’s test was not violated, the values were interpreted with sphericity. The results revealed a significant interaction between the factors with degrees of freedom (Df) (3, 15), an F value of 15.74, and a *p*-value less than 0.001. Furthermore, the partial eta squared was 0.759, and the observed power was 100%. Note that the model’s interaction between subject factors was insignificant (0.05).

Pearson’s correlation was conducted for trunk flexor and extensor strength; HGS; skinfold measures for biceps, triceps, subscapularis, and suprailiac; and fat percentage. A *p*-value less than 0.05 was considered statistically significant. A noticeable amount of association was found between trunk flexor and extensor strength (TF and TE), with Pearson’s (r) = 0.482, *p* = 0.002. A Pearson’s correlation showed a moderate association between trunk flexors and right HGS ((r) = 0.57, *p*-value *<* 0.001) as well as left HGS ((r) = 0.437, *p* = 0.005). Trunk extensor strength (TE) presented a moderate association with right HGS ((r) = 0.521, *p* = 0.001), while left HGS showed (r) = 0.553 and *p <* 0.001. Furthermore, the trunk’s flexor and extensor muscle strength showed a moderate positive association with overall HGS, presenting a Pearson’s (r) = 0.56 and 0.50, respectively. The fat percentage in the body and the thickness of the skinfold failed to prove an association with the trunk flexor and extensor muscle strength, yielding *p*-values greater than 0.05. The T score showed a moderate positive correlation with TE and TF (*p* = 0.562, *p* = 0.497), which was significant at *p* < 0.001. 

A multiple regression model was created to determine the relationships between the dependent variable TE and independent variables including waist measurement, age, and HGS. A stepwise method was employed. A significant regression was found, i.e., F (1, 37) = 16.33, with a *p*-value less than 0.001 and an R^2^ of 0.306. The participants’ dependent variable TE increased by 0.407 lbs with each kilogram/square meter increase in left-side HGS. The regression model was also created to predict the dependent variable TF, and the independent variables were waist measurement, age, and HGS. The stepwise method was used, and a significant regression was found, namely F (1, 38) = 17.84, *p <* 0.001, R^2^ 0.325. The participants’ predicted variable TF increased 0.362 pounds with each kilogram/square meter increase in HGS on the right side. Finally, right and left HGS were found to be predictors of trunk strength.

A multiple regression model was created to understand the relationship between the dependent variable TE and the independent variables T score, age, and height. The stepwise method was used. A significant regression was found, namely F (1, 37) = 17.11, with a *p*-value less than 0.001 and an R^2^ of 0.316. The participants’ dependent variable TE varied by 5.47 units with each unit variation in T score. A significant regression was found using the same predictor variable for TF: F (1, 37) = 12.13, with a *p*-value of 0.001 and an R^2^ of 0.247 for the independent variable T score. For the independent variable height, F (1, 36) = 12.13, with a *p*-value of 0.045 and an R^2^ of 0.247. The participants’ dependent variable TF varied by 3.37 units with each unit variation in T score, whereas the TF varied by 0.42 units with each unit variation in height. 

## 5. Discussion

HGS has been regarded as a new benchmark for nutritional status with direct implications for an individual’s health [7,8]. A weak HGS has a strong relationship with numerous unfavorable health results [25,26]. Previously published research reported an HGS of 61.1(10.5) kg among 25 men aged 20–29 years [24]. A HGS at or below 32 kg is considered weak in males [24]. Additionally, a weak HGS is linked with all-cause mortality and disability [27]. The study added a cut-off T score of −2 or below (equivalent to 32 kg in males or weaker) in the aging population [24]. The T score must be used in any research to stratify the population with a soft grip, as it may indicate an event in the prospective follow-up [27]. 

In the present study, the T-score values among the young participants were low (Table 3). A prominent finding was that among the young smokers in our sample, the T score was even lower. This finding should be further explored, as lifestyle changes have influenced the younger people in Saudi Arabia. A meta-analysis comparing T scores between the healthy aged population, the sarcopenic population, and the osteo-sarcopenic population found mean scores of 0.1 ± 0.04, −0.8 ± 0.2, and −2.9 ± 0.7, respectively, among males [25] older people. In comparison to the elderly populations included in various studies [25], T scores were lower among the younger population of Saudi Arabia [15]. The negative impact of smoking on HGS in this study corroborated similar observations in the previous literature [28]. 

The HGS predicted according to height was equivalent to the overall mean HGS (refer to Table 1) among the young university population. In the current study, we calculated the T score among the younger university population in reference to calculations used in a previous study [23,25]. We found that the muscle strength of the subjects varied in combination with several other risk factors in the healthy young participants. However, there is a need for the early use of the T score in diagnosing sarcopenia and osteoporosis among healthy adults [25]. The clinical utility of the “T score” suggests that it is a straightforward instrument that may assist clinicians in predicting poor physical health [29]. 

Moreover, the present study also found a positive correlation between T score and TE and TF. This association may also indicate a new relationship between T score and musculoskeletal issues, which can be identified early in adulthood. Some studies have explored the relationship between a low HGS over 50 years and common back problems [30]. Therefore, examining this association in a cohort of young adults is also necessary. The multiple regression models in the current study found a relationship between the T score and TE and TF. A unit change in the T score produced far greater changes in TE. However, current data on this finding are limited, and the issue should be explored further, as trunk strength is vital for functions of daily living, including the fundamental skills required to independently care for oneself, such as eating, bathing, step climbing, balance, and mobility [31]. 

On the contrary, skinfold thickness is a scientific measure that is often associated with hyperglycemia and insulin resistance among middle-aged persons [32]. Moreover, the unusual occurrence of insulin resistance in post-pubertal Asian Indian children has been associated with excess body fat, abdominal adiposity, and truncal subcutaneous fat [33]. 

There appeared to be minimal data on the relationship between body mass index, skinfold thickness, and cardiovascular disease risk factors among schoolchildren included in the Bogalusa Heart Study in the age group of 19 to 30 years [34]. However, the majority of participants in the current study were within the age group mentioned above. Hence, this age group is also an area for further research aiming to identify the population at high risk. In addition, researchers have found a strong relationship between skinfold thickness and cardiovascular disease (CVD) risk factors [34]. On the contrary, in the current study, no correlation was observed between skinfold thickness, TE, TF, and HGS. 

The association of HGS with the probability of mortality, cardio and related vascular disorders, cancer, renal failure, and premature deaths has been well-established in earlier research [8]. Currently, however, there are no data on the association between these factors and trunk strength, which is also an essential parameter for accomplishing daily living tasks. The statistical tools used in the present study established a moderate relationship, significant variation, and direct correlation between hand grip and trunk strength. Thus, trunk strength evaluation should also be a criterion for physical fitness to determine health status.

### Strength and Limitation

This study explored a unique association between HGS, SFT, and trunk strength. Future studies should explore a larger population sample using trunk extensor strength to create a valid database for people with various health conditions. Moreover, there were some limitations to the present research: due to COVID-19-related restrictions, we were not able to assess the full sample; however, it is worth noting that we included more than half of the sample size required for the study.

## 6. Conclusions

In clinical scenarios, the assessment of the trunk muscle strength is vital, as it can be used as a health indicator. In the present study, we found a moderate association between HGS, trunk muscle strength, and T score. However, no such association was observed for SFT at multiple sites. Additionally, a low T score for HGS was observed among the university students, which was further amplified by smoking as a risk factor. Hence, while planning a comprehensive evaluation, smoking should also be considered.

## Figures and Tables

**Table 1:** Demographic details of the participants.

Variable (*n* = 39)	Mean ± SD
Age (years)	21.59 ± 1.19
Smoking	Yes = 11 (28.2%) No = 28 (71.8%)
Dominance	Right = 37 Left = 2
Height (cm)	170.33 ± 6.07
Waist (cm)	76.02 ± 10.8
Body density	1.07 ± 0.01
Skinfold bicep measurement	6.15 ± 4.9
Skinfold triceps measurement	12.03 ± 6.2
Skinfold subscapularis measurement	13.14 ± 7.63
Hand grip strength right (kg/m^2^)	63.04 ± 13.35
Hand grip strength left (kg/m^2^)	58.34 ± 13.22
Hand grip strength overall (kg/m^2^)	59.62 ± 12.94
Predicted handgrip strength for height	59.63 ± 13.74
Trunk flexion strength (lbs)	50.95 ± 8.48
Trunk extension strength (lbs)	52.02 ± 9.72

kg/m^2^: kilograms per meter squared—SI unit for measurement of force; lbs: pounds—SI unit for measurement of force; cm: centimeters—SI unit for measurement of circumefrence.

**Table 2:** Distribution of participants in the study according to the body fat percentage.

Description	Frequency
Excellent	25
Good	10
Moderate	2
Overweight	2

**Table 3:** T scores of hand grip strength among the participants.

Age Group	Number of Participants	Known Factors	T Score			
			Mean	SD	Minimum	Maximum
19–24	39		0.0003	1	−1.95	2.5
	11	Smoking	−0.0186	0.98	−1.46	1.65
	28	Nonsmokers	0.0077	1.03	−1.95	2.15

A value less than or equal to −2 is considered low for people above 60 years [25].

## Data Availability

As the data belong to King Khalid University, disclosing the data publicly is restricted. Hence, the data will be made available only as per the university guidelines for internal data sharing.

## References

[1] Gale C.R., Martyn C.N., Cooper C., Sayer A.A. (2007). Grip strength, body composition, and mortality. Int. J. Epidemiol..

[2] Prasitsiriphon O., Pothisiri W. (2018). Associations of Grip Strength and Change in Grip Strength with All-Cause and Cardiovascular Mortality in a European Older Population. Clin. Med. Insights Cardiol..

[3] Vaidya S.M., Nariya D.M. (2021). Handgrip Strength as a Predictor of Muscular Strength and Endurance: A Cross-sectional Study. J. Clin. Diagn. Res..

[4] Bohannon R.W. (1997). Reference values for extremity muscle strength obtained by hand-held dynamometry from adults aged 20 to 79 years. Arch. Phys. Med. Rehabil..

[5] Pasquino A., Tomarchio A., De Cruto E., Conteduca J., Longo D., Russi V., Pica G., Meccariello L., Rollo G. (2021). Comparing hand strength and quality life of locking plate versus intramedullary k wire for transverse midshaft metacarpal fractures. Med. Glas (Zenica).

[6] Rantanen T. (2003). Muscle strength, disability and mortality. Scand. J. Med. Sci. Sports.

[7] Liu W., Leong D.P., Hu B., AhTse L., Rangarajan S., Wang Y., Wang C., Lu F., Li Y., Yusuf S. (2020). The association of grip strength with cardiovascular diseases and all-cause mortality in people with hypertension: Findings from the Prospective Urban Rural Epidemiology China Study. J. Sport Health Sci..

[8] Celis-Morales C.A., Welsh P., Lyall D.M., Steell L., Petermann F., Anderson J., Iliodromiti S., Sillars A., Graham N., Mackay D.F. (2018). Associations of grip strength with cardiovascular, respiratory, and cancer outcomes and all cause mortality: Prospective cohort study of half a million UK Biobank participants. BMJ.

[9] Supriya R., Singh K.P., Gao Y., Li F., Dutheil F., Baker J.S. (2021). A Multifactorial Approach for Sarcopenia Assessment: A Literature Review. Biology.

[10] Zaccagni L., Toselli S., Bramanti B., Gualdi-Russo E., Mongillo J., Rinaldo N. (2020). Handgrip Strength in Young Adults: Association with Anthropometric Variables and Laterality. Int. J. Environ. Res. Public Health.

[11] Doymaz F., Cavlak U. (2007). Relationship between thigh skinfold measurement, hand grip strength, and trunk muscle endurance: Differences between the sexes. Adv. Ther..

[12] Moffroid M.T. (1997). Endurance of trunk muscles in persons with chronic low back pain: Assessment, performance, training. J. Rehabil. Res. Dev..

[13] Wells J.C.K. (2005). Measuring body composition. Arch. Dis. Child..

[14] Asiri F., Dixit S., Alsubaie S.F., Gular K., Alshahrani A., Reddy R.S., Gautam A.P., Tedla J.S. (2022). Comparison of Neck Circumference, Waist Circumference, and Skinfold Thickness in Measuring the Subcutaneous Fat Distribution and Their Association with Handgrip Strength: Cross-Sectional Study. Int. J. Environ. Res. Public Health.

[15] Alrashdan A., Ghaleb A.M., Almobarek M. (2021). Normative Static Grip Strength of Saudi Arabia’s Population and Influences of Numerous Factors on Grip Strength. Healthcare.

[16] Centre of Disease Control and Prevention How to Protect Yourself & Others, 29 November 2021. https://www.cdc.gov/coronavirus/2019-ncov/prevent-getting-sick/prevention.html.

[17] World Health Organization (2011). Waist Circumference and Waist–Hip Ratio: Report of a WHO Expert Consultation: Ginebra, 8–11, 2011.

[18] Moreno L., Rodríguez G., Guillén J., Rabanaque M., León J., Ariño A. (2002). Anthropometric measurements in both sides of the body in the assessment of nutritional status in prepubertal children. Eur. J. Clin. Nutr..

[19] Alahmari K.A., Rengaramanujam K., Reddy R.S., Samuel P.S., Kakaraparthi V.N., Ahmad I., Tedla J.S. (2020). Cardiorespiratory Fitness as a Correlate of Cardiovascular, Anthropometric, and Physical Risk Factors: Using the Ruffier Test as a Template. Can. Respir. J..

[20] Durnin J.V.G.A., Womersley J. (1974). Body fat assessed from total body density and its estimation from skinfold thickness: Measurements on 481 men and women aged from 16 to 72 Years. Br. J. Nutr..

[21] Siri W.E. (1993). Body composition from fluid spaces and density: Analysis of methods. 1961. Nutrition.

[22] NIHR (2016). NIHR Southampton Biomedical Research Centre Procedure for Measuring No. June 2014.

[23] Yu R., Ong S., Cheung O., Leung J., Woo J. (2017). Reference Values of Grip Strength, Prevalence of Low Grip Strength, and Factors Affecting Grip Strength Values in Chinese Adults. J. Am. Med. Dir. Assoc..

[24] Dodds R.M., Syddall H.E., Cooper R., Benzeval M., Deary I.J., Dennison E.M., Der G., Gale C.R., Inskip H.M., Jagger C. (2014). Grip Strength across the Life Course: Normative Data from Twelve British Studies. PLoS ONE.

[25] Tarantino U., Greggi C., Visconti V., Cariati I., Tallarico M., Fauceglia M., Iundusi R., Albanese M., Chiaramonte C., Gasbarra E. (2021). T-Score and Handgrip Strength Association for the Diagnosis of Osteosarcopenia: A Systematic Review and Meta-Analysis. J. Clin. Med..

[26] Pratt J., De Vito G., Narici M., Segurado R., Dolan J., Conroy J., Boreham C. (2021). Grip strength performance from 9431 participants of the GenoFit study: Normative data and associated factors. Geroscience.

[27] Soysal P., Hurst C., Demurtas J., Firth J., Howden R., Yang L., Tully M.A., Koyanagi A., Ilie P.C., López-Sánchez G.F. (2020). Handgrip strength and health outcomes: Umbrella review of systematic reviews with meta-analyses of observational studies. J. Sport Health Sci..

[28] Saito T., Miyatake N., Sakano N., Oda K., Katayama A., Nishii K., Numata T. (2012). Relationship Between Cigarette Smoking and Muscle Strength in Japanese Men. J. Prev. Med. Public Health.

[29] Montalcini T., Migliaccio V., Yvelise F., Rotundo S., Mazza E., Liberato A., Pujia A. (2012). Reference values for handgrip strength in young people of both sexes. Endocrine.

[30] Park S.-M., Kim G.-U., Kim H.-J., Kim H., Chang B.-S., Lee C.-K., Yeom J.S. (2018). Low handgrip strength is closely associated with chronic low back pain among women aged 50 years or older: A cross-sectional study using a national health survey. PLoS ONE.

[31] Suri P., Kiely D.K., Leveille S.G., Frontera W.R., Bean J.F. (2009). Trunk Muscle Attributes Are Associated With Balance and Mobility in Older Adults: A Pilot Study. PM&R.

[32] Sievenpiper J.L., Jenkins D.J., Josse R.G., Leiter L.A., Vuksan V. (2001). Simple skinfold-thickness measurements complement conventional anthropometric assessments in predicting glucose tolerance. Am. J. Clin. Nutr..

[33] Misra A., Vikram N.K., Arya S., Pandey R.M., Dhingra V., Chatterjee A., Dwivedi M., Sharma R., Luthra K., Guleria R. (2004). High prevalence of insulin resistance in postpubertal Asian Indian children is associated with adverse truncal body fat patterning, abdominal adiposity and excess body fat. Int. J. Obes..

[34] Freedman D.S., Katzmarzyk P.T., Dietz W.H., Srinivasan S.R., Berenson G.S. (2009). Relation of body mass index and skinfold thicknesses to cardiovascular disease risk factors in children: The Bogalusa Heart Study. Am. J. Clin. Nutr..

